# Activation of LRP1 Ameliorates Cerebral Ischemia/Reperfusion Injury and Cognitive Decline by Suppressing Neuroinflammation and Oxidative Stress through TXNIP/NLRP3 Signaling Pathway in Mice

**DOI:** 10.1155/2022/8729398

**Published:** 2022-08-18

**Authors:** Cheng-Jie Yang, Xin Li, Xiao-Qing Feng, Ye Chen, Jian-Guo Feng, Jing Jia, Ji-Cheng Wei, Jun Zhou

**Affiliations:** ^1^Department of Anesthesiology, The Affiliated Hospital of Southwest Medical University, Luzhou, China; ^2^Laboratory of Anesthesiology, Southwest Medical University, Luzhou, China; ^3^Department of Traditional Chinese Medicine, The Affiliated Hospital of Southwest Medical University, Luzhou, China

## Abstract

Cerebral ischemia/reperfusion (I/R) injury is a clinical event associated with high morbidity and mortality. Neuroinflammation plays a crucial role in the pathogenesis of I/R-induced brain injury and cognitive decline. Low-density lipoprotein receptor-related protein-1 (LRP1) can exert strong neuroprotection in experimental intracerebral hemorrhage. However, whether LRP1 can confer neuroprotective effects after cerebral I/R is yet to be elucidated. The present study is aimed at investigating the effects of LRP1 activation on cerebral I/R injury and deducing the underlying mechanism involving TXNIP/NLRP3 signaling pathway. Cerebral I/R injury was induced in mice by bilateral common carotid artery occlusion. LPR1 ligand, apoE-mimic peptide COG1410, was administered intraperitoneally. To elucidate the underlying mechanism, overexpression of TXNIP was achieved via the hippocampal injection of AAV-TXNIP before COG1410 treatment. Neurobehavioral tests, brain water content, immunofluorescence, Western blot, enzyme-linked immunosorbent assay, HE, and terminal deoxynucleotidyl transferase dUTP nick end labeling staining were performed. Our results showed that the expressions of endogenous LRP1, TXNIP, NLRP3, procaspase-1, and cleaved caspase-1 were increased after cerebral I/R. COG1410 significantly ameliorated cerebral I/R-induced neurobehavioral deficits, brain edema, histopathological damage, and poor survival rate. Interestingly, COG1410 inhibited microglia proinflammatory polarization and promoted anti-inflammatory polarization, decreased oxidative stress, attenuated apoptosis, and inhibited the expression of the TXNIP/NLRP3 signaling pathway. However, the benefits of COG1410 were abolished by TXNIP overexpression. Thus, our study suggested that LRP1 activation with COG1410 attenuated cerebral I/R injury at least partially related to modulating microglial polarization through TXNIP/NLRP3 signaling pathway in mice. Thus, COG1410 treatment might serve as a promising therapeutic approach in the management of cerebral I/R patients.

## 1. Introduction

Cerebral ischemia/reperfusion (I/R) injury is a potentially life-threatening disorder with high morbidity and mortality [1, 2]. Primary ischemic insult is initiated due to insufficient oxygen and glucose delivery that supports cellular homeostasis [3]. On the other hand, restoring blood flow to the brain may cause a series of secondary pathological cascades, including excitotoxicity, neuroinflammation, oxidative stress, apoptosis, and death, or developing severe neurological deficits within the first several weeks due to delayed neuronal cell death [4, 5]. This phenomenon suggested that preventing and treating delayed neuronal necrosis are essential in addition to restoring cerebral blood flow in the treatment of cerebral I/R injury. However, a lack of specific neuroprotective agents for cerebral I/R injury necessitates the investigation of the underlying mechanism and finding effective therapeutic strategies.

Microglia are innate immune cells in the brain and homologous to macrophages in the peripheral system. Microglia play a vital role in the neuroinflammation of brain injury. Depending on the type of stimulus, activated microglia have distinct functions related to the M1/M2 microglial polarization [6]. The M1 phenotypes of microglia are proinflammatory, releasing tumor necrosis factor-a (TNF-*α*), interleukin-6 (IL-6), and inducible nitric oxide synthase (iNOS). Conversely, the M2 phenotypes of microglia are anti-inflammatory and conducive to the production of IL-10, transforming growth factor-*β* (TGF-*β*), and arginase-1 (Arg-1) [7]. Thus, microglial polarization modulation towards the anti-inflammatory M2 phenotype might be a promising therapeutic target, which ameliorates cerebral I/R injury.

Low-density lipoprotein receptor-related protein-1 (LRP1) is a multifunctional receptor with two main biological functions in endocytosis and in the regulation of cell signaling pathways, which is widely expressed in neurons and glial cells in the central nervous system (CNS) [8–10]. Apolipoprotein E (*APOE* = gene, apoE = protein), one of the ligands of LRP1, is synthesized by astrocytes and oligodendrocytes in the brain [11]. Human APOE consists of three different isoforms: APOE*ε*2, APOE*ε*3, and APOE*ε*4. Previous studies demonstrated that both human apoE3 and murine apoE exert neuroprotection by interacting with its receptor LRP1, while the APOEe4 isoform exhibits a poor prognosis in acute and chronic neurological diseases [12–14]. ApoE protein is 34 kilodaltons (kDa) and cannot permeate through the blood-brain barrier, making it difficult to use clinically. COG1410, a small molecule ApoE-mimetic peptide, is derived from the apoE receptor binding region [15]. Some studies showed that COG1410 has multiple neuroprotective properties, including anti-inflammation, antioxidation, and antiapoptosis, by activating its receptor LRP1 [16–18]. However, whether LRP1 activation with COG1410 can relieve I/R-induced cerebral damage and its underlying mechanism is yet unknown.

Nucleotide-binding oligomerization domain-like receptor protein 3 (NLRP3) inflammasome is composed of oligomerized NLRP3, apoptosis-associated speck-like protein (ASC), and procaspase-1 and is widely distributed in the microglia [19]. Previous studies found that NLRP3 inflammasomes promote inflammation and apoptosis by activating microglia in the cerebral I/R injury [20]. Moreover, oxidative stress is one of the crucial factors in cerebral I/R injury. Thioredoxin-interacting protein (TXNIP), an endogenous inhibitor of thioredoxin (TRX), plays a vital role in maintaining cellular redox balance [21, 22]. Previous studies demonstrated that TXNIP aggravates oxidative stress and promotes inflammatory response by activating the NLRP3 inflammasome. Therefore, TXNIP is also considered a potential target for the treatment of some I/R injuries [23, 24]. Nevertheless, the role of LRP1 and the TXNIP/NLRP3 signaling pathway in the microglial polarization modulation after cerebral I/R injury is yet to be elucidated.

In the present study, we hypothesized that activation of LRP1 with an apoE mimic, COG1410, alleviates cerebral I/R injury by regulating the polarization of microglia via inhibition of the TXNIP/NLRP3 signaling pathway.

## 2. Materials and Methods

### 2.1. Animals

The current study was approved by the Animal Care Committee of Southwest Medical University (China). Adult male *C57BL/6* mice, weighing 18–22 g, were purchased from Chengdu Dossy Experimental Animals Co., Ltd. (China). The animals were housed in cages in a temperature-controlled room (22 ± 2°C) with an alternating 12-h light/dark cycle and acclimatized for one week. Before the study, the mice were fasted for 8 h but were allowed water ad libitum. All procedures were performed according to the National Institutes of Health guidelines for the use of experimental animals.

### 2.2. Experimental Design

**Figure 1:**
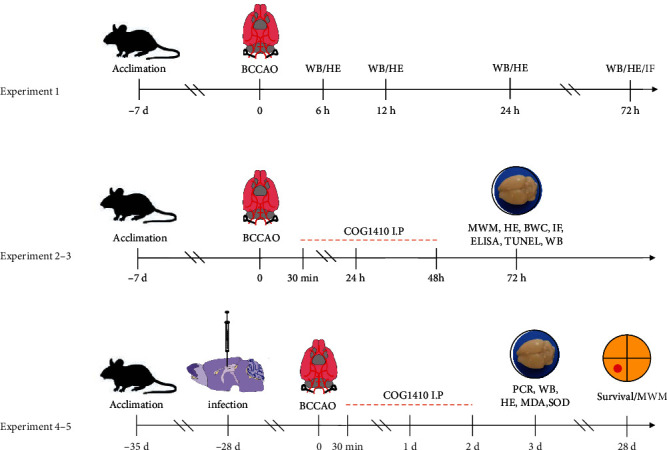
Experimental design and animal groups. BCCAO: bilateral common carotid artery occlusion; WB: Western blot; HE: hematoxylin and eosin; IF: immunofluorescence; BWC: brain water content; MWM: Morris water maze; ELISA: enzyme-linked immunosorbent assay; TUNEL: terminal deoxynucleotidyl transferase dUTP nick end labeling; h: hour; d: day.

The experimental flow diagram is illustrated in Figure 1.

#### 2.2.1. Experiment 1

To evaluate the time course of endogenous LRP1, TXNIP, NLRP3, procaspase-1, and cleaved caspase-1 expression and histopathological changes in the hippocampal tissues of mice after cerebral I/R, the mice were randomized into five groups: sham, 6, 12, 24, and 72 h after I/R (*n* = 14/group). Western blot analysis was performed to assess the protein level of LRP1, TXNIP, NLRP3, procaspase-1, and cleaved caspase-1. Then, hematoxylin and eosin (HE) staining was used for histopathological examination of the hippocampal tissues. Additionally, the cellular localization of LRP1 with neurons (neuronal nuclei, NeuN), astrocytes (glial fibrillary acidic protein, GFAP), or microglia (calcium-binding adaptor molecule 1, Iba-1) was evaluated by double immunofluorescence staining in the sham and I/R-72 h group (*n* = 3/group).

#### 2.2.2. Experiment 2

To determine the beneficial effects of LRP1 activation on neurobehavioral functions, brain water content (BWC), and histopathological changes at 72 h after cerebral I/R, the mice were randomized into five groups: sham, I/R + Vehicle, I/R + COG1410 (0.5 mg/kg), I/R + COG1410 (1.0 mg/kg), and I/R + COG1410 (2.0 mg/kg) (*n* = 24/group). The BWC and histopathological changes were tested at 72 h after reperfusion, while Morris water maze (MWM) was performed for neurobehavior tests on days 3–8 after cerebral I/R.

#### 2.2.3. Experiment 3

To assess the roles of LRP1 activation on microglia, neuroinflammation, and apoptosis at 72 h after cerebral I/R, the mice were randomly divided into three groups for Western blot, enzyme-linked immunosorbent assay (ELISA), immunofluorescence, and terminal deoxynucleotidyl transferase dUTP nick end labeling (TUNEL) staining: sham, I/R + Vehicle, and I/R + COG1410 (*n* = 14/group).

#### 2.2.4. Experiment 4

In order to investigate the underlying mechanism of TXNIP/NLRP3 pathway in LRP1 mediated microglia polarization-modulating, the mice were randomized into five groups: sham, I/R + Vehicle, I/R + COG1410, I/R + COG1410 + AAV-NC, and I/R + COG1410 + AAV-TXNIP (*n* = 14/group). Histopathological changes, the level of oxidative stress, the release of inflammatory factors, and Western blot analysis were performed at 72 h after cerebral I/R. Moreover, the mice were randomly divided into three groups, naïve, naïve + AAV-NC, and naïve + AAV-TXNIP (*n* = 6/group), to verify the efficacy of AAV-TXNIP on TXNIP overexpression via qRT-PCR and Western blot.

#### 2.2.5. Experiment 5

To explore the underlying mechanism of TXNIP/NLRP3 pathway in LRP1-mediated long-term neuroprotective effect, the mice were randomly divided into five groups: sham, I/R + Vehicle, I/R + COG1410, I/R + COG1410 + AAV-NC, and I/R + COG1410 + AAV-TXNIP (*n* = 15/group). The survival rates of different groups were monitored at 28 days after cerebral I/R, and WMW was conducted on days 28–33 after cerebral I/R.

### 2.3. Cerebral I/R Model

The model of cerebral I/R was established using bilateral common carotid artery occlusion (BCCAO) as described previously [25]. Briefly, mice were anesthetized with pentobarbital sodium (45 mg/kg, intraperitoneally). Then, the trachea was exposed by a 0.5 cm midline neck incision, and the bilateral common carotid arteries (CCAs) were separated from the vagus nerve. Subsequently, the bilateral CCAs were occluded with the microvascular clamp, with the right CCA clamped first. After 30 min of ischemia, both CCAs were reperfused. In the sham operation group mice, the same procedures were performed without CCA occlusion. After infiltration with 0.25% bupivacaine, the wound was closed by sterile sutures. During the surgery, the body temperature of mice was maintained with a heating pad.

### 2.4. Intraperitoneal Administration

COG1410 (acetyl-AS-Aib-LRKL-Aib-KRLL-amide synthesized by GL Biochem, Shanghai, China) was diluted with saline to different concentrations (0.5 mg/kg, 1.0 mg/kg, and 2.0 mg/kg), as described previously (Laskowitz et al., 2007). A volume of 100 *μ*L COG1410 was administered intraperitoneally at 30 min, 24 h, and 48 h after reperfusion. The mice in the sham and I/R + Vehicle groups received equal volumes of saline at the same time point.

### 2.5. AAV Injection

The TXNIP overexpression mouse model was established on day 28 before BCCAO, as described previously [26]. Briefly, the adeno-associated virus vectors (AAV-TXNIP and AAV-NC) were constructed by Syngentech Co., Ltd. (Beijing, China). Before exposing the skull, the mice were anesthetized with pentobarbital sodium in a stereotaxic apparatus (RWD, Shenzhen, China). A volume of 1.5 *μ*L of the AAV vectors was injected stereotaxically into the bilateral hippocampal CA1 area (AP-2.0 mm; ML±1.0 mm; DV 2.0 mm) at a rate of 0.5 *μ*L/min, following which the needle was kept in place for 5 min and screwed out slowly. The skin incision was sutured, and the mice were housed in a single cage after they awoke from anesthesia.

### 2.6. Specimen Collection

The mice were sacrificed at 6, 12, 24, or 72 h after reperfusion under deep anesthesia. Some animals were perfused transcardially with ice-cold saline, and then, the hippocampal tissues were separated on ice, frozen in liquid nitrogen, and stored at -80°C for Western blot, ELISA, detection of oxidative stress, and RT-PCR. The other mice were perfused transcardially with 4% paraformaldehyde for HE, TUNEL, and immunofluorescence staining.

### 2.7. Morris Water Maze Test

The function of spatial learning and memory for mice was assessed using the Morris water maze (MWM) test [27]. The maze (Xinruan, Shanghai, China) consisted of a round tank (height 50 cm, diameter 120 cm), a platform (diameter 10 cm), and a camera analysis system. The tank was divided into four quadrants, and filled up to a depth of 30 cm with mixed milk water (22 ± 1°C). The mice were trained for four trials per day for five consecutive days during the place navigation test. In every trial, mice were placed in a different start quadrant and allowed to find the platform submerged below 1 cm of the water surface within a maximum duration of 60 s; the escape latency was recorded. On day 6, in the probe trial, the platform was withdrawn, and the mice were allowed to navigate freely in water for 60 s. The number of times crosses over the removed platform, duration in the target quarter (%), and swimming speed was recorded during the trial.

### 2.8. Histopathological Examination

Whole-brain tissues were fixed in 4% paraformaldehyde, embedded in paraffin, and sliced into 3 *μ*m-thick coronal sections. The sections were stained with HE and observed under a light microscope (Olympus, Tokyo, Japan) by two experienced pathologists blinded to the study. The number of normal neurons per square millimeter (cells/mm^2^) was calculated according to the size of the CA1 subregion in four high-magnification fields of each section, and the average was considered.

### 2.9. Brain Water Content

The brain water content (BWC) was estimated by the wet-to-dry brain weight ratio as described previously [28]. Briefly, the whole brains were harvested under deep anesthesia and immediately weighed to obtain the wet weight. Then, the brain tissues were baked at 65°C for 48 h to obtain the dry weight. BWC = (wet weight − dry weight)/wet weight × 100%.

### 2.10. Assessment of Apoptosis

The brains were dehydrated with 30% sucrose and sliced into 8 *μ*m-thick coronal frozen sections using a cryostat (Leica, Germany). The apoptosis of cells in the hippocampus was detected using the TUNEL staining, according to the manufacturer's protocol (#12156792910, Roche, Basel, Switzerland) [29]. The numbers of TUNEL-positive cells were counted in the CA1 subregion of the hippocampus. Four random high-magnification fields per section over a microscopic field of 400x magnification were averaged. Data were presented as the ratio of TUNEL-positive cells (%).

### 2.11. Immunofluorescence Staining

Immunofluorescence staining was performed as described previously [30]. Briefly, the frozen sections were incubated with primary antibody at 4°C overnight: mouse anti-Iba1 (1 : 200, #GT10312, Invitrogen, USA), mouse anti-GFAP (1 : 200, #3670S, Cell Signaling Technology (CST), USA), mouse anti-NeuN (1 : 200, #ab104224, Abcam, USA), rabbit anti-LRP1 (1 : 200, #64099S, CST, USA), rabbit anti-TXNIP (1 : 200, #14715S, CST, USA), and rabbit anti-NLRP3 (1 : 200, #15101S, CST, USA). After washing with phosphate-buffered saline (PBS), the sections were incubated with the corresponding secondary antibody (Cy3-labeled goat anti-rabbit IgG, FITC-labeled goat anti-mouse IgG) (1 : 200, #S0011, #0007, Affinity, USA) at room temperature for 1 h, followed by DAPI (#C0060, Solarbio, China) staining. Finally, the sections were visualized, and images were captured using a fluorescence microscope.

### 2.12. Detection of Inflammatory Factors

Hippocampal tissue homogenates were prepared and clarified by centrifugation at 12000 rpm at 4°C for 15 min. TNF-*α*, IL-6, IL-1*β*, and IL-18 levels were measured in the supernatants of hippocampal tissues using the ELISA kits (#MM-0132M1, #MM-0163M1, #MM-0040M1, #MM-0169M1, Meimian Jiangsu, China) by the manufacturer's instruction as described previously [31].

### 2.13. Detection of Oxidative Stress

Hippocampal tissue homogenates were prepared, and lysates were collected by centrifugation at 3000 rpm at 4°C for 10 min. The ROS and MDA content and SOD and GSH-Px activity in the supernatant of hippocampal tissues were detected by commercial biochemical kits (#E004-1-1, #A003-1-2, #A001-3-2, #A005-1-2, Nanjing Jiancheng, China) as described previously [32, 33].

### 2.14. qRT-PCR

Total RNA was extracted from the hippocampal tissues using an RNA simple total RNA kit (#DP419, Tiangen, Beijing, China) following the standard protocol. This isolated RNA was reverse-transcribed into cDNA using the ReverTra Ace qPCR RT Master Mix (#FSQ-201, Toyobo, Japan) according to the manufacturer's protocol. Then, the cDNA was amplified using SuperReal PreMix Plus (SYBR Green) (#FP205-02, Tiangen, Beijing, China) on a real-time PCR system (Roche). The amplification parameters were as follows: 95°C for 15 min, followed by five cycles of 95°C for 10 s and 60°C for 32 s. *β*-Actin was used as an internal reference for the quantification of the *TXNIP* gene expression level. The relative mRNA expression level in the naive group (target mRNA/*β-actin* value) was set as the normalization to calculate the fold-changes of the mRNA level in other groups. The primer sequences are listed in Table 1.

### 2.15. Western Blot Analysis

The protein in the hippocampal tissue sample was estimated using an enhanced BCA protein assay kit (#P0012, Beyotime, Shanghai, China). An equivalent of 30 *μ*g protein was separated on the sodium dodecyl sulfate-polyacrylamide gel electrophoresis (SDS-PAGE) and transferred to a polyvinylidene fluoride membrane (PVDF; Amersham Biosciences, NJ, USA) or a nitrocellulose membrane (Beyotime). Then, the membrane was blocked with 5% nonfat milk at room temperature for 1 h and probed with the primary antibodies at 4°C overnight: anti-LRP1 (1 : 1000, #64099S, CST), anti-TXNIP (1 : 1000, #14715S, CST), anti-NLRP3 (1 : 1000, #15101S, CST), anti-caspase-1 (1 : 200, #SC-56036, Santa Cruz, USA), anti-Iba-1 (1 : 1000, #17198S, CST), anti-iNOS (1 : 1000, #AF0199, Affinity, USA), anti-Arg-1 (1 : 1000, #DF6657, Affinity), anti-Bcl-2 (1 : 1000, #ab59348, Abcam, USA), anti-Bax (1 : 1000, #50599-2-Ig, Proteintech, China), anti-cleaved caspase-3 (1 : 1000, #19677-1-AP, Proteintech), and anti-*β*-actin (1 : 5000, #6609-1-Ig, Proteintech), followed by incubation with the appropriate horseradish peroxidase- (HRP-) labeled secondary antibody (1 : 5000, #SE131, #SE134, Solarbio, China) at room temperature for 1 h. Then, the membranes were developed using enhanced chemiluminescence (ECL; Amersham, Buckinghamshire, UK). Finally, the immunoreactive bands were analyzed using Image J software (version 1.31; National Institutes of Health (NIH), Bethesda, MD, USA).

### 2.16. Statistical Analysis

The GraphPad Prism 8.3 statistical software (GraphPad Software, San Diego, CA, USA) was used for statistical analysis. All data were expressed as the mean and standard error of the mean (mean ± SEM). One-way analysis of variance (ANOVA) and Tukey's post hoc test were used for comparisons. For the training phase of the WMW test, the escape latency over time was analyzed by two-way repeated-measures ANOVA. Kaplan-Meier survival curves were analyzed using the log-rank test. *P* < 0.05 was considered statistically significant.

## 3. Results

### 3.1. Time Course of Histopathological Changes and the Endogenous Protein Levels of LRP1, TXNIP, and NLRP3 Inflammasome in the Hippocampus after Cerebral I/R

**Figure 2:**
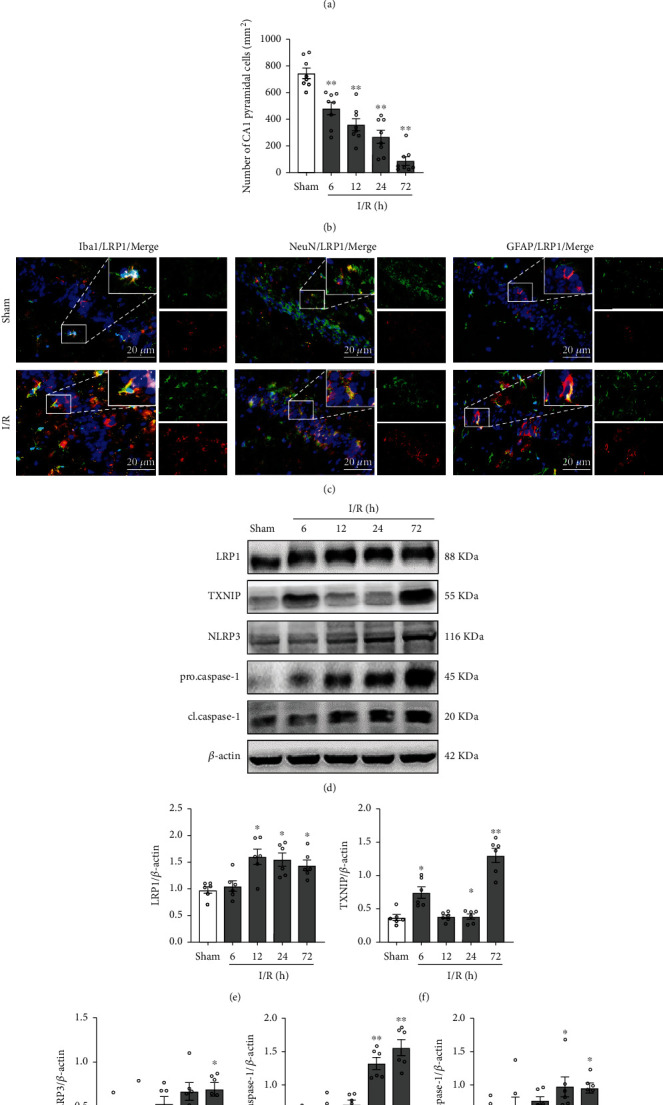
Effects of cerebral I/R on histopathological changes, and the expressions of LRP1, TXNIP, and NLRP3 inflammasome. (a) HE staining of hippocampal CA1 area (×400), scale bar = 20 *μ*m; the arrows indicate injured neurons. (b) Quantitative analysis of normal neurons in the hippocampal CA1 area. (c) Double immunofluorescence staining (IF) of LRP1 (red) with microglia (Iba-1, green), neurons (NeuN, green), and astrocytes (GFAP, green) in the hippocampal CA1 region at 72 h after cerebral I/R. Nuclei were stained with DAPI (blue), scale bar = 20 *μ*m. (d) Representative bands of Western blot data. (e–i) Quantitative analysis of the Western blot bands. Data were represented as mean ± SEM. Western blot, *n* = 6 per group; HE staining, *n* = 8 per group; double immunofluorescence staining, *n* = 3 per group. ^∗^*P* < 0.05, ^∗∗^*P* < 0.01 vs. sham group.

HE staining was performed to determine the histopathological changes in the hippocampal tissues on the animal models. The results showed that the total normal cells of the CA1 region started decreasing at 6 h and were the lowest at 72 h after cerebral I/R injury (Figures 2(a) and 2(b)). Furthermore, Western blot analysis showed that the protein level of LRP1 was significantly increased in the I/R group compared to the sham group at 12-72 h after surgery. The protein level of TXNIP was significantly increased in the I/R group compared to the sham group at 6 h and 72 h postsurgery. The protein level of NLRP3 was significantly increased in the I/R group compared to the sham group at 72 h after surgery. The protein level of procaspase-1 and cleaved caspase-1 was significantly increased in the I/R group compared to the sham group at 24-72 h after surgery (Figures 2(d)–2(i)).

Double immunofluorescence staining of LRP1 with microglia (Iba-1), neurons (NeuN), and astrocytes (GFAP) was performed in the sham group and I/R-72 h group. The cellular colocalization of LRP1 with Iba-1, NeuN, and GFAP was detected in the CA1 subregion of the hippocampus, and the number of LRP1-positive microglia, neurons, and astrocytes was increased at 72 h after cerebral I/R (Figure 2(c)).

### 3.2. COG1410 Ameliorated Short-Term Neurological Deficits, Brain Edema, and Histopathological Damage at 72 H after Cerebral I/R

**Figure 3:**
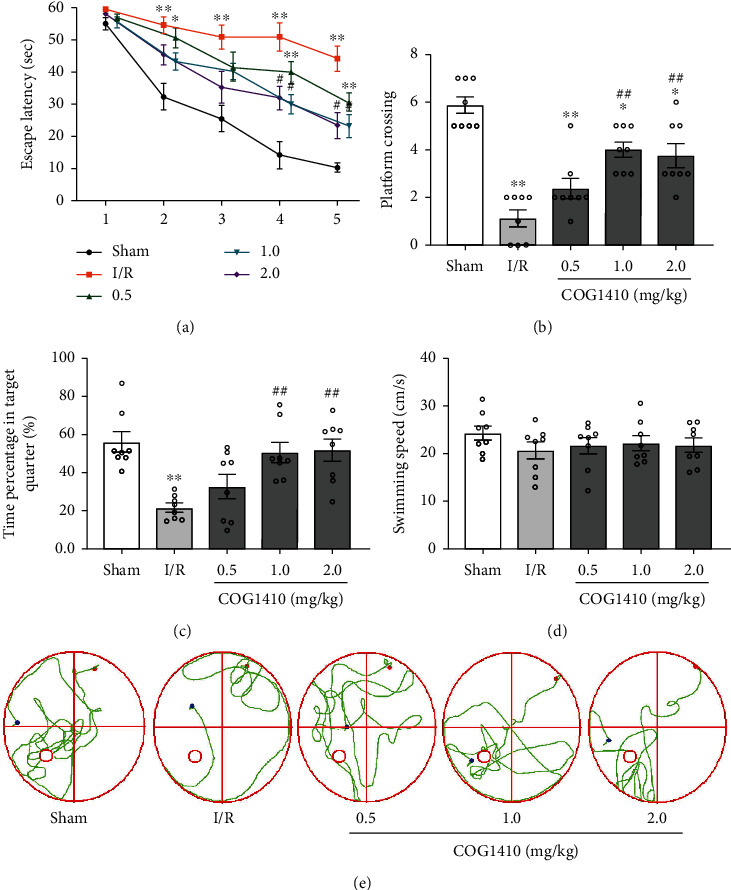
Effects of COG1410 on the neurological functions at 72 h after cerebral I/R. (a) Escape latency of mice in the training trials. (b) Frequency of platform crossing in the probe trial. (c) Percentage of time spent in the target quadrant in the probe trial. (d) Swimming speed in the probe trial. (e) Swimming trajectory of mice of each group in the probe trial. Data were represented as mean ± SEM (*n* = 8). ^∗^*P* < 0.05, ^∗∗^*P* < 0.01 vs. sham group; ^#^*P* < 0.05, ^##^*P* < 0.01 vs. I/R + Vehicle group.

In the place navigation test, the escape latency for the mice to find the platform was significantly prolonged in the I/R + Vehicle group compared to the sham group. However, a significantly shorter escape latency was observed on training days 4-5 in the I/R + COG1410 (1.0 mg/kg and 2.0 mg/kg) groups (Figure 3(a)). In the probe trial, the mice in the I/R + Vehicle group required fewer platform crossings and less time percentage in the target quarter compared to the sham group, while the reference memory deficits were improved by COG1410 treatment (1.0 mg/kg and 2.0 mg/kg). Also, no significant differences were observed in the swimming speed among each group (Figures 3(b)–3(e)).

**Figure 4:**
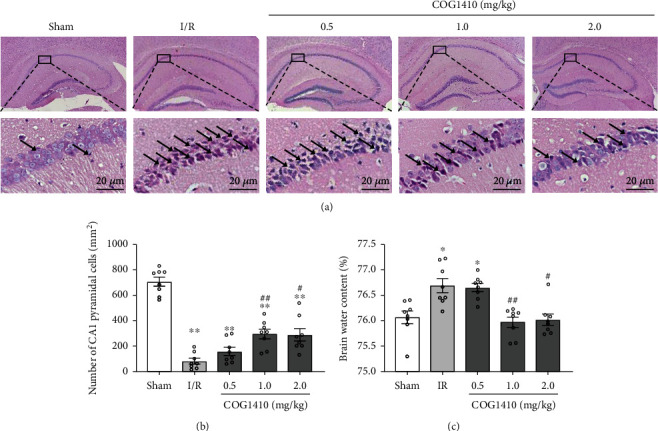
Effects of COG1410 on the histopathological damage and BWC at 72 h after cerebral I/R. (a) HE staining of hippocampal CA1 area (×400), scale bar = 20 *μ*m; the arrows indicate injured neurons. (b) Quantitative analysis of normal neurons in the hippocampal CA1 area. (c) BWC. Data were represented as mean ± SEM (*n* = 8). ^∗^*P* < 0.05, ^∗∗^*P* < 0.01 vs. sham group; ^#^*P* < 0.05, ^##^*P* < 0.01 vs. I/R + Vehicle group.

The results of HE staining showed that the structure of the hippocampal CA1 region was regular morphology in the sham group. However, many injured neurons, characterized by shrunken and stained dark, were observed in the hippocampal CA1 region of the mice after cerebral I/R. Moreover, fewer normal neurons of the hippocampal CA1 area and higher BWC of the whole brain tissues were observed in the I/R + Vehicle group compared to the sham group mice at 72 h after cerebral I/R. Compared to the animals treated with vehicles, administration of 1.0 mg/kg and 2.0 mg/kg COG1410 significantly attenuated the histopathological damage and brain edema (Figures 4(a)–4(c)). Based on the MWM test, histopathological results, and BWC, the dose of 1.0 mg/kg was selected for subsequent experiments.

### 3.3. COG1410 Reduced Apoptosis of Hippocampal Cells at 72 H after Cerebral I/R

**Figure 5:**
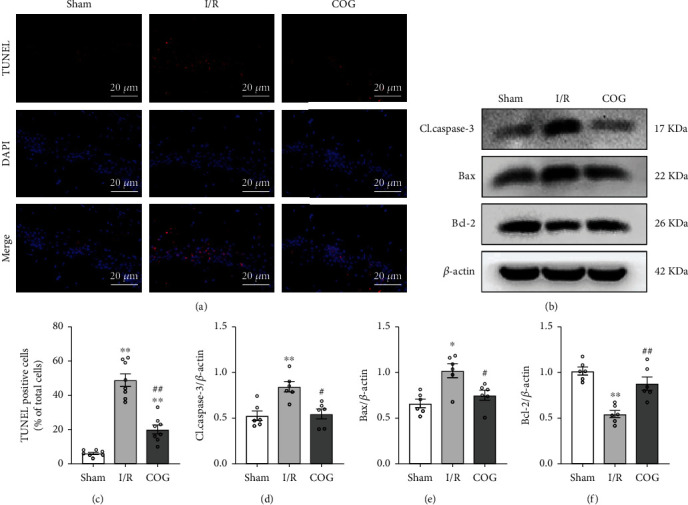
Effects of COG1410 on hippocampal apoptosis at 72 h after cerebral I/R. (a) Representative images of TUNEL staining in the hippocampal CA1 area (×400); nuclei were labeled with blue fluorescence (DAPI), and TUNEL-positive cells were labeled with red fluorescence, scale bars = 20 *μ*m. (b) Representative bands of Western blot data. (c) Quantitative analyses of TUNEL-positive cells in the hippocampal area. (d–f) Quantitative analysis of the Western blot bands. Data were represented as mean ± SEM. Western blot, *n* = 6 per group; TUNEL staining, *n* = 8 per group. ^∗^*P* < 0.05, ^∗∗^*P* < 0.01 vs. sham group; ^#^*P* < 0.05, ^##^*P* < 0.01 vs. I/R + Vehicle group.

The cell apoptosis of the hippocampal CA1 region in mice was evaluated by the TUNEL method. Apoptotic cells were not detected in the sham group. The number of apoptotic cells was significantly increased in the hippocampal CA1 region after 72 h of reperfusion. These changes were alleviated by COG1410 treatment, manifested as a decreased number of TUNEL-positive cells (Figures 5(a) and 5(c)).

Furthermore, the Western blot data indicated that the protein levels of cleaved caspase-3 and Bax increased in the I/R + Vehicle group compared to the sham group, while that decreased significantly in the I/R + COG group compared to the I/R + Vehicle group (Figures 5(b), 5(d), and 5(e)). In addition, the protein levels of Bcl-2 were significantly lower in the I/R + Vehicle group compared to the sham group but were increased in the I/R + COG group compared to the I/R + Vehicle group (Figures 5(b) and 5(f)).

### 3.4. COG1410 Promoted M1-to-M2 Phenotypic Polarization of Microglia and Alleviated Inflammatory Response

**Figure 6:**
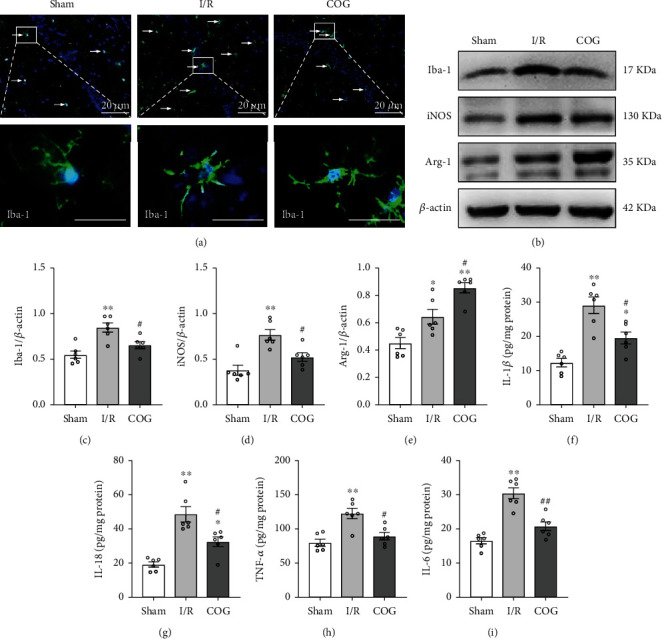
Effects of COG1410 on microglial polarization and proinflammatory factors at 72 h after cerebral I/R. (A) Representative images of Iba-1 immunofluorescence staining in hippocampal CA1 area (×200); nuclei were labeled with blue fluorescence (DAPI), and Iba-1 was labeled with green fluorescence, scale bars = 20 *μ*m. (b) Representative bands of Western blot data. (c–e) Quantitative analysis of the Western blot bands. (f–i) Quantitative analysis of the inflammatory factors including IL-1*β*, IL-18, TNF-*α*, and IL-6. Data were represented as mean ± SEM. Western blot and ELISA, *n* = 6 per group; immunofluorescence staining, *n* = 3 per group. ^∗^*P* < 0.05, ^∗∗^*P* < 0.01 vs. sham group; ^#^*P* < 0.05, ^##^*P* < 0.01 vs. I/R + Vehicle group.

Immunofluorescence staining with Iba-1 antibody was performed at 72 h after cerebral I/R. The sham group showed fewer Iba-1-positive cells with ramified thin processes. However, compared to the sham group, the number of Iba-1-positive cells was significantly increased, and the microglial cells showed considerable branch retraction, thickening, and amoeba-like morphology in the I/R + Vehicle group, while the I/R + COG group showed less microglial activation than the I/R + Vehicle group (Figure 6(a)).

Upon activation, microglial polarized to M1 or M2 phenotypes with distinct proinflammatory and immunosuppressive functions, among which iNOS was an M1 phenotype marker and Arg-1 was an M2 phenotype marker. The Western blot data indicated that the protein levels of Iba-1 and iNOS were upregulated in the I/R + Vehicle group compared to the sham group but were significantly lower in the I/R + COG group compared to the I/R + Vehicle group (Figures 6(b)–6(d)). Moreover, the protein level of Arg-1 was increased in the I/R + Vehicle group compared to the sham group and was significantly higher in the I/R + COG group compared to the I/R + Vehicle group (Figures 6(b) and 6(e)).

Next, we detected the expression of inflammatory factors, including IL-1*β*, IL-18, TNF-*α*, and IL-6 in each group by ELISA. As shown in Figures 6(f)–6(i), the levels of inflammatory factors were significantly increased in the I/R + Vehicle group compared to the sham group, while a significantly decreased expression of the inflammatory factors was observed in the I/R + COG group compared to the I/R + Vehicle group.

### 3.5. COG1410 Inhibited TXNIP/NLRP3 Signaling Pathway in the Hippocampus after Cerebral I/R

**Figure 7:**
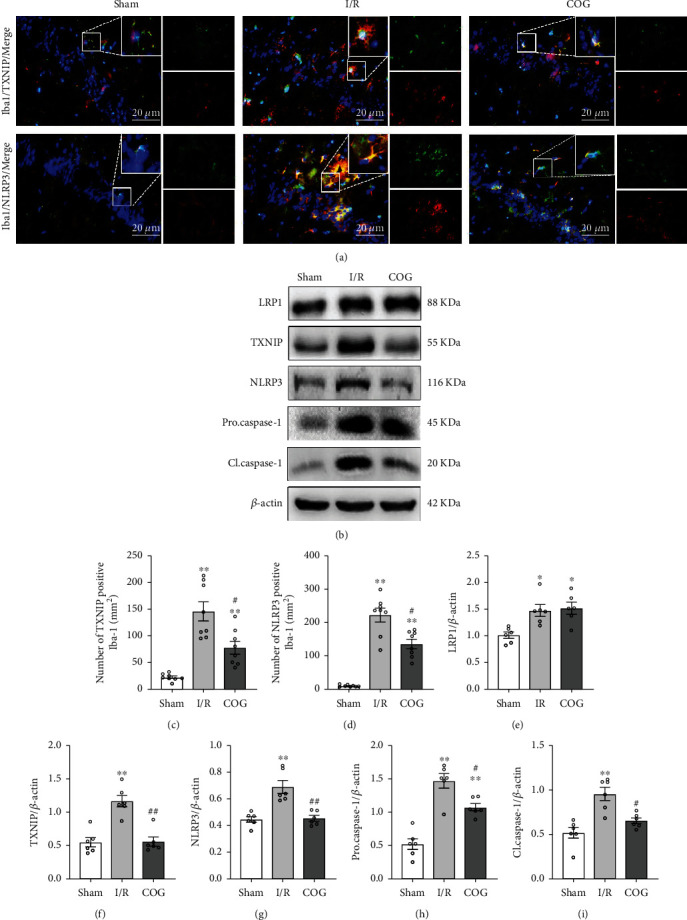
Effects of COG1410 on the TXNIP/NLRP3 signaling pathway at 72 h after cerebral I/R. (a) Representative images of the colocalization of TXNIP and NLRP3 (red) with microglia (Iba-1, green) in the hippocampal area. (b) Representative bands of Western blot data. (c, d) Quantitative analysis of TXNIP-positive and NLRP3-positive microglia. (e–i) Quantitative analysis of the Western blot bands. Data were represented as mean ± SEM. Western blot, *n* = 6 per group; double immunofluorescence staining, *n* = 8 per group. ^∗^*P* < 0.05, ^∗∗^*P* < 0.01 vs. sham group; ^#^*P* < 0.05, ^##^*P* < 0.01 vs. I/R + Vehicle group.

Western blot was used to detect the levels of LRP1, TXNIP, NLRP3, procaspase-1, and cleaved caspase-1 in the hippocampus of mice at 72 h after cerebral I/R. The results showed that the levels of these proteins increased in the I/R + Vehicle group compared to the sham group. Interestingly, the expression levels of TXNIP, NLRP3, procaspase-1, and cleaved caspase-1 decreased significantly in the I/R + COG group compared to the I/R + Vehicle group. However, no statistically significant difference was observed between the I/R + Vehicle group and the I/R + COG group concerning the LRP1 level (Figures 7(b) and 7(e)–7(i)).

Double immunofluorescence staining was performed to verify the cellular localization of the TXNIP/NLRP3 signaling pathway after cerebral I/R. The results showed that TXNIP/NLRP3 signaling pathway was colocalized with microglia in the hippocampus of mice at 72 h after cerebral I/R. Moreover, quantitative analysis showed that the number of TXNIP-positive and NLRP3-positive microglia increased significantly in the I/R + Vehicle group compared to the sham group, while the number of the TXNIP-positive and NLRP3-positive microglia was significantly decreased in the I/R + COG group compared to the I/R + Vehicle group (Figures 7(a), 7(c), and 7(d)).

### 3.6. Overexpression of TXNIP Reversed the Effects of COG1410 on M2 Microglial Polarization, Apoptosis, Oxidative Stress, and Inflammation

**Figure 8:**
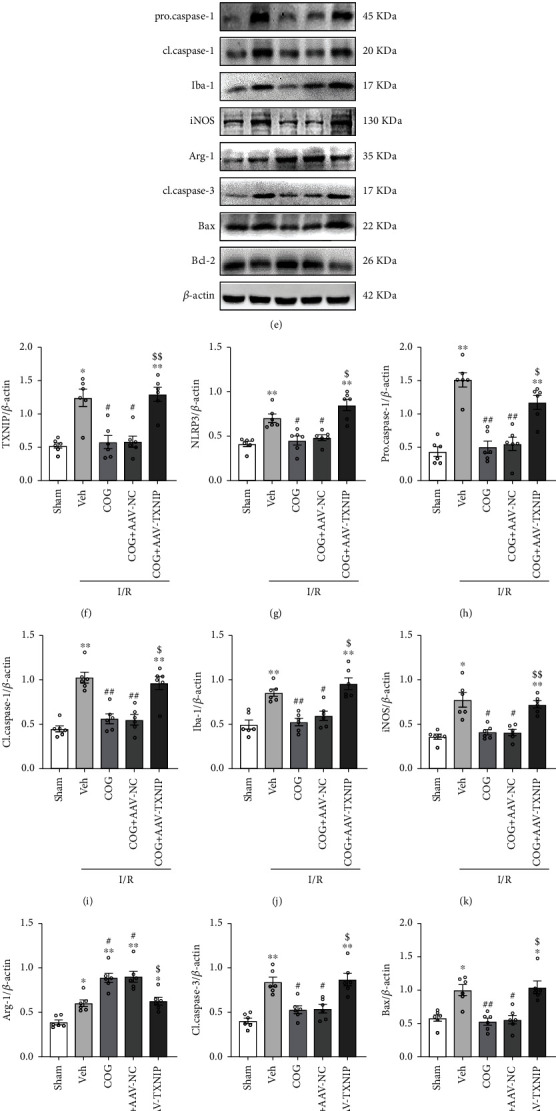
Overexpression of TXNIP reversed the beneficial effects of COG1410 on M2 microglial polarization and apoptosis at 72 h after cerebral I/R. (a–c) TXNIP-overexpression efficacy evaluation. Data were represented as mean ± SEM (*n* = 6). ^∗^*P* < 0.05, ^∗∗^*P* < 0.01 vs. naive group; ^#^*P* < 0.05, ^##^*P* < 0.01 vs. AAV-NC group. (e) Representative bands of Western blot data. (d, f–o) Quantitative analysis of the Western blot bands. Data were represented as the mean ± SEM (*n* = 6). ^∗^*P* < 0.05, ^∗∗^*P* < 0.01 vs. sham group; ^#^*P* < 0.05, ^##^*P* < 0.01 vs. I/R + Vehicle group; ^$^*P* < 0.05, ^$$^*P* < 0.01 vs. I/R + COG1410 + AAV-NC group.

AAV vectors with green fluorescence were injected into the hippocampus 28 days before BCCAO. The overexpression efficacy of AAV-TXNIP was verified by Western blot and qRT-PCR. The results showed that both the mRNA and protein levels of TXNIP were significantly increased in the AAV-TXNIP group compared to the AAV-NC group (Figures 8(a)–8(c)).

Strikingly, compared to vehicle treatment after cerebral I/R, COG1410 treatment had no effect on the protein level of LRP1, but it significantly decreased the protein levels of TXNIP, NLRP3, procaspase-1, cleaved caspase-1, M1 microglial phenotype marker Iba-1 and iNOS, and proapoptotic proteins cleaved caspase-3 and Bax and increased the levels of M2 microglial phenotype marker Arg-1 and antiapoptotic protein Bcl-2. When the TXNIP protein was overexpressed in the I/R + COG + AAV-TXNIP group, the effects of COG1410 on the TXNIP/NLRP3 signaling pathway inhibition, M2 microglial polarization, and antiapoptosis were reversed compared to the I/R + COG + AAV-NC group (Figures 8(d)–8(o)).

**Figure 9:**
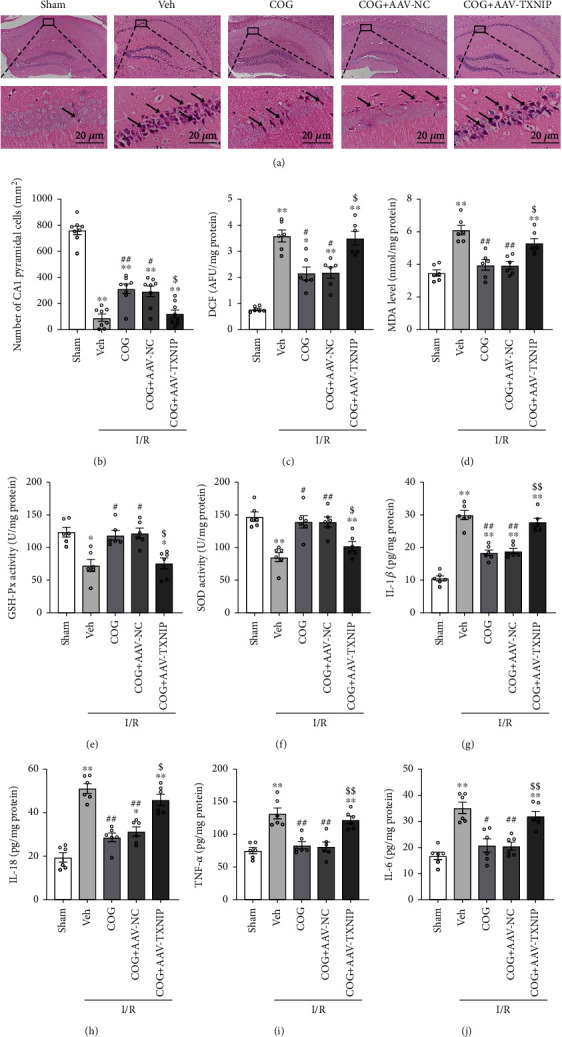
Overexpression of TXNIP reversed the beneficial effects of COG1410 on histopathological damage, oxidative stress, and inflammation at 72 h after cerebral I/R. (a) HE staining of hippocampal CA1 area (×400), scale bar = 20 *μ*m; the arrows indicate injured neurons. (b) Quantitative analysis of normal neurons in the hippocampal CA1 area. (c–f) Quantitative analysis of the oxidative stress markers (ROS, MDA, GSH-Px, and SOD). (g–j) Quantitative analysis of the inflammatory factors including IL-1*β*, IL-18, TNF-*α*, and IL-6. Data were represented as mean ± SEM. HE staining, *n* = 8 per group; oxidative stress markers and inflammatory factors, *n* = 6 per group. ^∗^*P* < 0.05, ^∗∗^*P* < 0.01 vs. sham group; ^#^*P* < 0.05, ^##^*P* < 0.01 vs. I/R + Vehicle group; ^$^*P* < 0.05, ^$$^*P* < 0.01 vs. I/R + COG1410 + AAV-NC group.

The results of HE staining showed that the normal neuronal cells in the hippocampus were significantly increased in the I/R + COG group compared to the I/R + Vehicle group and that in the I/R + COG + AAV-TXNIP group was also decreased compared to the I/R + COG + AAV-NC group (Figures 9(a) and 9(b)). Since TXNIP plays a vital role in maintaining the balance of cellular redox, we assessed the degree of oxidative stress via ROS and MDA content and GSH-Px and SOD activity and found that the degree of oxidative stress was significantly reduced with COG1410 treatment after cerebral I/R. Nevertheless, the antioxidative effect of COG1410 was abolished when the TXNIP protein was overexpressed (Figures 9(c)–9(f)). Meanwhile, we found that the levels of inflammatory factors, including IL-1*β*, IL-18, TNF-*α*, and IL-6, were significantly reduced with COG1410 treatment after cerebral I/R. However, the anti-inflammatory effect of COG1410 was abolished when the TXNIP protein was overexpressed (Figures 9(g)–9(j)).

### 3.7. COG1410 Improved Survival Rate and Long-Term Neurological Functions through the TXNIP/NLRP3 Signaling Pathway after Cerebral I/R

**Figure 10:**
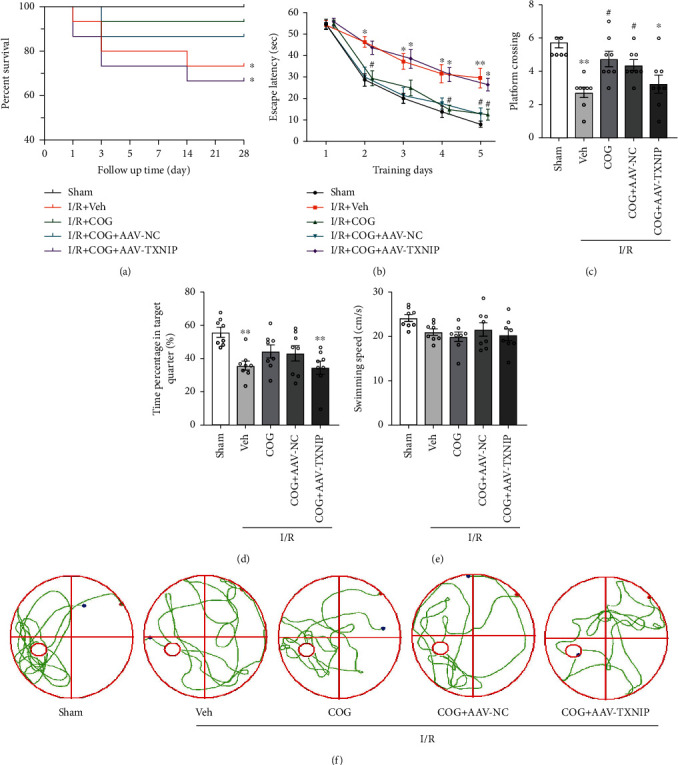
COG1410 improved the survival rate and long-term neurological function 28 days after cerebral I/R. (a) The survival rate in different groups. (b) Escape latency of mice in the training trials. (c) Frequency of platform crossing in the probe trial. (d) Percentage of time spent in the target quadrant in the probe trial. (e) Swimming speed in the probe trial. (f) Swimming trajectory of mice of each group in the probe trial. Data were represented as mean ± SEM. Survival rate, *n* = 15 per group; MWM, *n* = 8 per group. ^∗^*P* < 0.05, ^∗∗^*P* < 0.01 vs. sham group; ^#^*P* < 0.05, ^##^*P* < 0.01 vs. I/R + Vehicle group.

To evaluate the long-term effects of COG1410 on cerebral I/R mice, a 28-day survival rate for different groups was observed. Interestingly, the survival rate was significantly decreased in the I/R + Vehicle group compared to the sham group, while that of the cerebral I/R mice was improved by COG1410 treatment. However, the effect of COG1410 on improving the survival rate of cerebral I/R mice was abolished after overexpression of TXNIP (Figure 10(a)). The results of the MWM test showed that compared to the sham group, the learning and memory function were impaired in the mice 28 days after cerebral I/R. The neurological deficits of cerebral I/R mice were alleviated with COG1410 treatment, while the neuroprotective function of COG1410 was reversed by TXNIP overexpression (Figures 10(b)–10(f)).

## 4. Discussion

In the current study, we first investigated the beneficial effects of LRP1 activation and explored its underlying mechanism in the cerebral I/R mouse model. Next, we demonstrated that LRP1 activation with COG1410 regulated microglia polarization, decreased brain edema, reduced oxidative stress, suppressed apoptosis in the hippocampus, and improved both short- and long-term neurological functions after cerebral I/R. In addition, LRP1 activation was associated with the downregulation of TXNIP, NLRP3, procaspase-1, cleaved caspase-1, Iba-1, iNOS, IL-1*β*, IL-18, IL-6, TNF-*α*, cleaved caspase-3, Bax, ROS, and MDA and the upregulation of Arg-1, Bcl-2, GSH-Px, and SOD after cerebral I/R. However, overexpression of TXNIP reversed the beneficial effects of LRP1 activation on microglial polarization, inflammation, oxidative stress, apoptosis, and neurological functions. These results suggested that LRP1 activation attenuates neuroinflammation, apoptosis, and oxidative stress after cerebral I/R, which was, at least partially, related to microglial polarization mediated by the inhibition of the TXNIP/NLRP3 signaling pathway.

In the present study, a BCCAO-induced cerebral I/R mice model was used. The hippocampal neurons are related to the functions of spatial learning and memory, of which the CA1 neurons are most vulnerable to ischemic damage. Transient BCCAO delays hippocampal neuron death and is often used to study the mechanism of cerebral I/R injury [34]. However, the BCCAO model shows great variability due to the presence of posterior communicating arteries (PcomA). Previous studies have confirmed that C57BL/6 mice have less PcomA patency, which is ideal for establishing a BCCAO animal model [35–37]. In this study, HE staining was used to determine the success of the mice model. The results of the histopathological analysis showed that the number of normal hippocampal pyramid cells significantly decreased 72 h after BCCAO, which was consistent with previous studies [38, 39].

The Morris water maze experiment, as a classic experiment for mouse behavioral evaluation, is divided into two parts: the positioning navigation experiment and the space exploration experiment [28, 29]. In this study, the Morris water maze experiment was performed on days 3-8 after cerebral I/R. The positioning navigation experiment was carried out on days 3-7 after cerebral I/R (training days 1-5), and the space exploration experiment was carried out on days 8 after cerebral I/R. The positioning navigation experiment evaluates the learning ability, and the space exploration experiment evaluates the memory ability. Considering that learning is a process, when all mice are in the initial learning phase, they cannot show differences in neurobehavioral deficits. Therefore, it is speculated that there are two possible reasons for the difference between the Morris water maze experiment and the activation of LRP1/TXNIP/NLRP3. Firstly, the neurobehavioral deficits might have appeared on day 3 after cerebral I/R (training day 1), while the mice among different groups were undergoing training in the positioning navigation experiment at this time, so there were no differences in escape latency. Secondly, the expressions of the LRP1/TXNIP/NLRP3 pathway in this experiment were markedly increased at 3 days after cerebral I/R injury; however, they might be further upregulated on days 4-5 after cerebral I/R (training days 2-3). This might be consistent with the changing trend in the results of neurobehavioral deficits.

Microglia, a hallmark of neuroinflammation, plays a critical role in the delayed neuronal death caused by cerebral I/R. Activated microglia causes endocytosis of cell debris and participates in tissue repair in the early stage of cerebral I/R. However, overactivated brain microglia release inflammatory factors, such as TNF-*α* and IL-6, and participate in chronic inflammation, causing delayed neuronal death, brain edema, and neurological deficits [40, 41]. In this study, the results of double immunofluorescence staining showed that the LRP1 was colocalized with microglia, neurons, and astrocytes in the CA1 subregion of the hippocampus at 72 h after cerebral I/R. Previous study indicated that direct protein-protein binding effects of apoE with its signal receptor LRP1 enable microglia to exert immunosuppressive and neuroprotective effects in neuroinflammation caused by hemorrhagic stroke, traumatic brain injury, and multiple sclerosis [42]. Another study has shown that activation of LRP1 with COG1410 promotes microglial M1 to M2 phenotypic shift, and LRP1 was expressed in M2 phenotypes but not in M1 phenotypes of activated microglia in subarachnoid hemorrhage (SAH) mice [43]. In the present study, we found that the activation of LRP1 by COG1410 significantly decreased the iNOS-positive M1 phenotypes but increased the Arg-1 positive M2 phenotypes of activated microglia. Moreover, apoE-mimic peptide COG1410 treatment significantly inhibited neuroinflammation, reduced apoptosis in the hippocampal CA1 area, decreased brain edema, and improved short- and long-term neurological function after cerebral I/R in mice.

Furthermore, how LRP1 regulates microglial M1 to M2 phenotypic shift after cerebral I/R is yet to be elucidated. The potential mechanism involves the cleavage of the intracellular domain of the LRP1 after interacting with its ligand. The cytoplasmic fragment of LRP1 may have functions in the cytoplasm or the nucleus via protein cascades or transcriptional regulation [44]. A previous study showed that the C-terminal fragment of LRP1 regulates microglial activation via inhibited JNK phosphorylation after the binding between apoE-mimic peptide COG1410 and LRP1 [45]. Another study found that stimulation of LRP1 by apoE-mimic peptide COG1410 modulated microglial polarization through Shc1/PI3K/Akt signaling pathway [43]. Nonetheless, the mechanisms of LRP1 underlying microglial phenotypic shift in cerebral I/R are yet to be elucidated. TRX is a vital intracellular antioxidant substance that exists as TRX/TXNIP under physiological conditions. The generation of a large number of reactive oxygen species (ROS) increases the expression of TXNIP and activates the NLRP3 inflammasome during the postischemic reperfusion phase [46].

LRP1 is a transmembrane receptor composed of a transmembrane *β* chain of 85 kDa noncovalently to an extracellular *α* chain of 515 kDa. It mainly exerts its biological functions in two ways of endocytosis and signal transduction. At present, the specific mechanism of LRP1 signal transduction remains to be elucidated. Previous studies have demonstrated that different motifs in the intracellular domain of the LRP1 *β* chain (LRP1-ICD) have different functions: YXXL motifs are related to endocytosis, while NPXY motifs can bind to various proteins in the cytoplasm (such as DAB1, FE65, JIP1, PSD-95, ShcA, and CED-6/GULP). When LRP1 binds to its ligand, LRP1-ICD of the LRP1 *β* chain can be cleaved through regulated intramembranous proteolysis (RIP), and the released fragments can play a protein cascade role in the cytoplasm or translocate to the nucleus to regulate transcription [4–7]. In this experiment, we found that the expression of TXNIP decreased after the cerebral I/R mice were treated with the LRP1 agonist COG1410. Interestingly, the expression of LRP1 did not change significantly for the cerebral I/R mice that TXNIP was overexpressed. It is speculated that LRP1 acts as an upstream signal of TXNIP and regulates the expression of TXNIP through the protein cascade or transcriptional regulation of LRP1-ICD.

As an important antioxidant in cells, TRX has two types: TRX1 and TRX2. TRX1 is distributed in the cytoplasm, nucleus, and plasma membrane, while TRX2 is distributed in the mitochondria. TXNIP is an endogenous inhibitor of TRX and exists as the composition TRX/TXNIP complex under physiological conditions. During oxidative stress, an increase in ROS leads to a decrease in TRX in the nucleus, accompanied by free TXNIP increases and translocates to the cytoplasm and mitochondria, where it binds TRX1 and TRX2, respectively, resulting in an increase in apoptosis signal-regulating kinase 1 (ASK-1) and causing apoptosis and aggravate oxidative stress. At the same time, TXNIP can directly bind to NLRP3 and activate the NLRP3 inflammasome, further aggravating oxidative stress [22]. Previous studies have also demonstrated that the regulation of NLRP3 by TXNIP is dependent on ROS [47, 48]. In this study, it was found that the decrease and increase of TXNIP correspond to the decrease and increase of oxidative stress, respectively. It is speculated that the increased ROS during oxidative stress activates TXNIP/NLRP3 pathway and activated NLRP3 inflammasome will further aggravate oxidative stress and form a vicious cycle.

Activation of the NLRP3 inflammasome appears to occur in two steps. The first step involves priming or initiating a signal in which many PAMPs or DAMPs are recognized by TLRs, leading to activation of nuclear factor kappa B- (NF-*κ*B-) mediated signaling, which in turn upregulates transcription of inflammasome-related components, including inactive NLRP3, proIL-1*β*, and proIL-18. The second step of inflammasome activation is the oligomerization of NLRP3 and subsequent assembly of NLRP3, ASC, and procaspase-1 into a complex. This triggers the transformation of procaspase-1 to caspase-1, as well as the production and secretion of mature IL-1*β* and IL-18. To date, several stimuli (such as ATP, ROS, cell debris, silica, asbestos, amyloid-*β*, and alum) have been found as the activator of the second step of inflammasome activation. Meanwhile, a variety of NLRP3-inflammasome activators including ATP, ROS, and silica were shown to result in TXNIP dissociation from TRX and binding to the NLRP3 inflammasome. TXNIP binding is an essential step for NLRP3 inflammasome activation. On the other hand, some studies demonstrated that the NLRP3 inflammasome and its downstream IL-1*β* activate the microglial M1 phenotypes [49, 50]. The activated M1 microglia, in turn, promote inflammation and oxidative stress, thus forming a vicious circle [51, 52]. Therefore, the TXNIP/NLRP3 signaling pathway is speculated to play a major role in regulating the polarization of microglia. The current study found that microglial M1 phenotypes are elevated, accompanied by TXNIP, NLRP3, procaspase-1, and cleaved caspase-1 upregulation after cerebral I/R, while increased microglial M2 phenotypes are accompanied by TXNIP, NLRP3, procaspase-1, and cleaved caspase-1 downregulation after COG1410 treatment. In addition, the results of double immunofluorescence staining showed that the number of TXNIP-positive and NLRP3-positive microglia were increased in the hippocampus of mice after cerebral I/R, while these types of cells were decreased after COG1410 treatment. Also, hippocampal apoptosis induced by cerebral I/R was observed, as indicated by increased positive cells in TUNEL staining, upregulated proapoptotic proteins cleaved caspase-3 and Bax, and downregulated anti-apoptotic protein Bcl-2. Previous studies have shown that cleaved caspase-1 catalyzed by activated NLRP3 inflammasomes promotes the mature release of IL-1*β* and IL-18 to regulate inflammation and apoptosis [53]. Some studies also found that TXNIP promotes apoptosis by activating proapoptotic protein signal-regulating kinase (ASK-1) and inducing the release of cytochrome-C from mitochondria [54]. Therefore, it could be deduced that apoptosis is also significantly reduced following the inhibition of the TXNIP/NLRP3 signaling pathway by activating LRP1 in this study.

To further verify the correlation between LRP1 and the TXNIP/NLRP3 signaling pathway, the TXNIP protein was overexpressed through the hippocampal injection of AAV vectors in this study. TXNIP overexpression with AAV-TXNIP significantly reversed the effects of LRP1 activation on TXNIP/NLRP3 pathway inhibition, M2 microglial polarization, antiapoptosis, anti-inflammatory, and antioxidant, while the LRP1 levels remained unchanged. These data suggested that LRP1 activation promotes M2 microglial polarization by inhibiting the TXNIP/NLRP3 pathway after cerebral I/R in mice.

Nevertheless, the present study had some limitations. Firstly, we only found that the activation of LRP1 affects the TXNIP/NLRP3 signaling pathway; however, the specific mechanism needs further study. Secondly, apoE is bound and internalized via receptor-mediated endocytosis by some receptors, such as the low-density lipoprotein receptor (LDLR), apoE receptor 2 (apoEr2), and triggering the receptor expressed on myeloid cells 2 (TREM2) in the CNS. How the apoE-mimic peptide COG1410 affects these receptors and the related pathways need to be investigated further. Thirdly, we did not fully explore the mechanism by which NLRP3 modulates microglia/macrophage polarization. A previous study has demonstrated that curcumin protected against ischemic stroke by modulating microglia/macrophage polarization toward an anti-inflammatory phenotype through NF-*κ*B suppression and NLRP3 inflammasome inhibition. Meanwhile, NLRP3 knockdown in vivo significantly inhibited microglia/macrophage pyroptosis and ameliorated cerebral ischemia injury [55]. Further research has shown that MiR-423-5p could decrease the polarization of microglia/macrophage cells of the M1 phenotype by inhibiting the mRNA expression of NLRP3 in a spinal cord injury animal model [56]. Another study also found that Tranilast, an NLRP3 inflammasome inhibitor, could promote the polarization of microglia/macrophage from M1 type to M2 type and ameliorate ischemic stroke by inhibiting the NLRP3/caspase-1 pathway in a murine model of distal middle cerebral artery occlusion [57]. The activation of the NLRP3 inflammasome triggers the transformation of procaspase-1 to caspase-1, as well as the production and secretion of mature IL-1*β* and IL-18. Interestingly, some research supported a potential role for IL-1*β* as one of the potential supporting factors of M1 over M2 microglia/macrophage polarization. Administration of an IL-1*β* neutralizing antibody led to upregulation of the M2 marker, Ym1, and corresponding downregulation of the proinflammatory cytokine, cleaved IL-1*β* in LPS-activated BV2 microglia cell [58]. Likewise, our study found that inhibition of NLRP3 inflammasome promotes the polarization of microglia from M1 type to M2 type. The current study found that administration of COG1410 led to downregulation of the expressions of TXNIP, NLRP3, caspase-1, IL-1*β*, the M1 markers iNOS, and TNF-*α*, while the M2 marker Arg-1 was up-regulated in cerebral I/R injury mice. It is speculated that COG1410 could regulate microglia phenotype polarization through TXNIP/NLRP3/caspase-1/IL-1*β* pathway. At the same time, inflammation is a multimolecular cascade, and hence, the possibility of involvement of multiple pathway regulation is likely and requires further study. Finally, the clinical translation of this study needs to be further elucidated.

## 5. Conclusions

**Figure 11:**
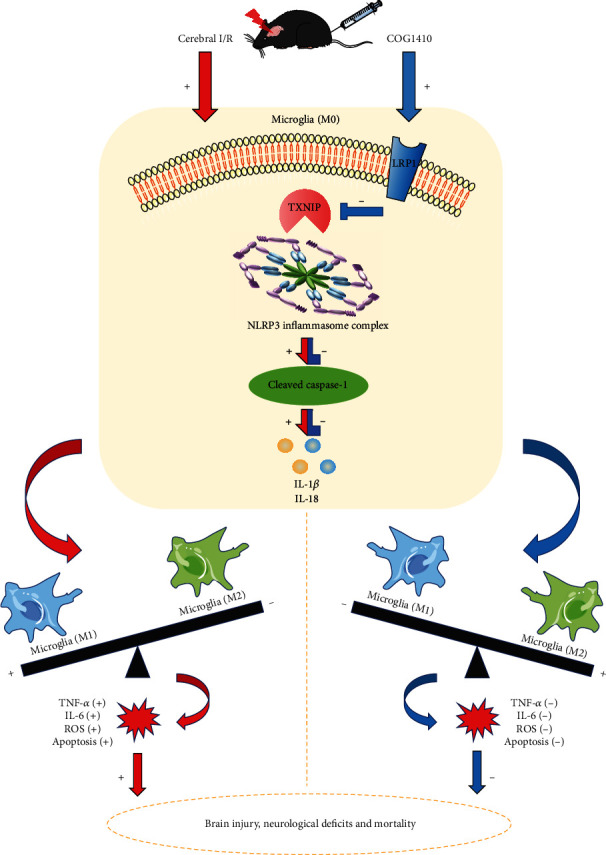
The mechanism map of the protective properties of COG1410 against cerebral I/R injury. Inflammation, oxidative stress, and apoptosis were triggered by cerebral I/R, which resulted in brain injury, neurological deficits, and even mortality. COG1410 treatment could improve the changes mentioned above. Mechanistically, COG1410 of LRP1 agonist shifted microglia towards the M2 phenotype by inhibiting the TXNIP/NLRP3 signaling pathway. The activation was labeled with “+,” and the inhibition was labeled with “-.”

This study demonstrated that LRP1 activation by apoE-mimic peptide COG1410 attenuated neuroinflammation, apoptosis, and oxidative stress and could improve short- and long-term neurological deficits and mortality after cerebral I/R, which was, at least in part, related to microglial polarization mediated by the inhibition of TXNIP/NLRP3 signaling pathway (Figure 11). Therefore, apoE-mimic peptide COG1410 may be a promising treatment agent in patients after cerebral I/R injury.

## Figures and Tables

**Table 1:** The PCR primers sequences.

Genes	Primer sequences
TXNIP	Forward: 5′-ATACTCCTTGCTGATCTACG-3′
	Reverse: 5′-TGGGGTATCTGGGATGTTTA-3′

*β*-Actin	Forward: 5′-TTTGCAGCTCCTTCGTTGC-3′
	Reverse: 5′-TCGTCATCCATGGCGAACT-3′

Abbreviation: TXNIP: thioredoxin-interacting protein.

## Data Availability

The data used to support the findings of this study are included within the article and are available from the corresponding author upon request.
